# Putative Systemic Biomarkers of Biomass Smoke-Induced Chronic Obstructive Pulmonary Disease among Women in a Rural South Indian Population

**DOI:** 10.1155/2018/4949175

**Published:** 2018-11-22

**Authors:** Sangeetha Vishweswaraiah, Tania Ahalya Thimraj, Leema George, Chaya Sindaghatta Krishnarao, Komarla Sundararaja Lokesh, Jayaraj Biligere Siddaiah, Kjell Larsson, Swapna Upadhyay, Lena Palmberg, Mahesh Padukudru Anand, Koustav Ganguly

**Affiliations:** ^1^SRM Research Institute, SRM Institute of Science and Technology (Formerly SRM University), Chennai 603203, India; ^2^Department of Pulmonary Medicine, JSS Medical College and Hospital, JSS Academy of Higher Education and Research, Mysuru, India; ^3^Work Environment Toxicology, Institute of Environmental Medicine, Karolinska Institutet, Box 287, SE-171 77 Stockholm, Sweden

## Abstract

**Rationale:**

Exposure to biomass smoke (**BMS**) has been implicated in chronic obstructive pulmonary disease (**COPD**). About 3 billion people worldwide use biomass fuel for cooking and heating. Women in rural communities of low- and lower-middle-income countries are disproportionately exposed to massive amounts of BMS during active cooking hours (4–6 h/day). Therefore, BMS exposure is considered as a risk factor for COPD in the same order of magnitude as tobacco smoke. In rural India, due to cultural reasons, women are the primary cook of the family and are mostly nonsmokers. Thus, BMS-induced COPD is predominant among rural Indian women. However, BMS-COPD remains a relatively unexplored health problem globally. Therefore, we investigated the serum chemokine and cytokine signatures of BMS-COPD and tobacco smoke-induced COPD (**TS-COPD**) patients compared to their control in a rural South Indian population for this field study.

**Methods:**

Concentrations of 40 serum chemokines and cytokines were measured using a multiplexed immunoassay. The study cohort consisted of **BMS-COPD** (female; *n* = 29) and BMS-exposed subjects without COPD (**BMS-CONTROL**; female; *n* = 24). For comparison, data from TS-COPD patients (male, *n* = 23) and tobacco smokers without COPD (**TS-CONTROL**; male, *n* = 22) were investigated. Subjects were matched for age, sex, and biomass exposure. Tobacco consumption was slightly higher in TS-COPD subjects compared to TS-CONTROL. BMS-exposed and TS-exposed subjects (currently exposed) were from the same locality with similar dwelling habits and socioeconomic status. A validated structured questionnaire-based survey and spirometry was performed. An additional control group with no tobacco and BMS exposure (**TS-BMS-CONTROL**; *n* = 15) was included. Statistical significance was set at *p* ≤ 0.01.

**Results:**

Serum median concentrations (pg/ml) of **CCL15** [8799.35; 5977.22], **CCL27** [1409.14; 1024.99], and **CXCL13** [37.14; 26.03] were significantly higher in BMS-CONTROL compared to BMS-COPD subjects. Nine analytes exhibited higher concentrations in TS-CONTROL compared to TS-COPD subjects. Comparison of chemokine and cytokine concentrations among BMS-COPD versus TS-COPD and BMS-CONTROL versus TS-CONTROL subjects also revealed distinct molecular signatures.

**Conclusion:**

Our data identifies CCL27 and CXCL13 as putative, plausibly homeostatic/protective biomarkers for BMS-COPD within the investigated population that warrants validation in larger and multiple cohorts. The findings further indicate exposure-specific systemic response of chemokines and cytokines.

## 1. Introduction

Biomass smoke (**BMS**) exposure is considered as a global risk factor for chronic obstructive pulmonary disease (**COPD**) development in the same order of magnitude as tobacco smoke. There are an estimated 3 billion solid fuel users as against 1.1 billion tobacco smokers worldwide [1, 2] (http://www.who.int/gho/tobacco/use/en/; http://www.who.int/mediacentre/factsheets/fs292/en/). Approximately 50% of all households globally and 90% of rural households in low- and lower-middle-income countries continue to use biomass fuel as their main source of domestic energy [1]. In 2015 alone, 3.2 million deaths were due to COPD and it accounted for 2.6% of global disability adjusted life years (DALYs) [3]. Currently, COPD is ranked as the 3rd leading cause of death worldwide (http://www.who.int/mediacentre/factsheets/fs310/en/). About 90% of people suffering from and succumbing to COPD belong to low and lower-middle-income countries [2]. However, till date most COPD research has been carried out in high-income countries with a focus on tobacco smoking as the main cause [2]. Therefore, it is evident that COPD as a whole and in particular BMS-induced COPD remains an under-appreciated and under-researched health topic in low- and lower-middle-income countries including India [4].

Biomass smoke constitutes an important fraction of household air pollution [5]. The World Health Organization (WHO) reported about 4.3 million global deaths in 2012 due to household air pollution (http://www.who.int/mediacentre/factsheets/fs292/en/). About 50% COPD deaths in developing countries are attributable to BMS, of which ~ 75% are women [6, 7]. In countries like India, women in rural areas are exposed to massive amounts of BMS while cooking (4–6 h/day) in poorly ventilated dwellings for about 30–40 years during their lifetime. Due to sociocultural reasons, women in rural India are the primary cooks of the family and are mostly nonsmokers. Correspondingly, the tobacco-smoking population of rural India is mainly men. Conservative estimates suggest that there are about 30 million COPD patients in India at present [8, 9]. The ratio of male to female COPD patients in India is 1.5 : 1.0, whereas the male-to-female smoker's ratio is 10 : 1 [10]. Therefore, it is evident that biomass smoke-induced COPD (**BMS-COPD**) is predominant among rural Indian women [11] whereas tobacco smoke-induced COPD (**TS-COPD**) is predominant among Indian men. Due to the above exposure scenario, the current study is focused on women for the BMS-exposed subgroup and men for the smoking subgroup.

Biomass fuel refers to plant- and/or animal-based materials that are burnt for energy. It includes wood and charcoal, twigs, grass, or agricultural crop residues and dried animal dung (e.g., cow dung) [5, 12, 13]. Biomass smoke contains respirable particulate matter (PM_2.5_ and PM_10_), carbon monoxide, oxides of nitrogen and sulfur, benzene, formaldehyde, 1,3-butadiene, polycyclic aromatic hydrocarbons, free radicals, aldehydes, volatile organic compounds, chlorinated dioxins, oxygenated and chlorinated organic matter, and endotoxin [5, 13]. Almost 100 times higher PM_10_ levels (20,000 *μ*g/m^3^) have been recorded in households using biomass fuel with poor ventilation compared to WHO and Environmental Protection Agency (EPA) limits [5, 13].

The pathophysiology of TS-COPD and BMS-COPD is different. BMS-COPD exhibits disproportionately greater bronchial involvement, less emphysema, and higher prevalence of respiratory failure, pulmonary hypertension, and airway hyperinflation than to TS-COPD [5, 14–17]. These observations suggest that the phenotype of COPD may be related to specific prototypes of environmental/extrinsic exposures, which in turn implicate exposure-specific molecular patho-mechanisms. Altered host defense is a hallmark of COPD that is characterized by local (lung) as well as systemic alterations of cytokine and chemokine regulation [18]. In view of the severe paucity of data on BMS-COPD in India [4, 19] and worldwide, we compared the serum chemokine and cytokine signatures of BMS-COPD and TS-COPD patients to their respective controls. Moreover, identification of putative systemic biomarkers that may aid in prevention, diagnosis, and defining therapeutic strategies is of wider clinical significance [20]. Our study included a validated structured questionnaire (demographics, exposure, and respiratory symptoms) and pulmonary function measurements among subjects residing in the rural areas of Nanjangud subdistrict, Mysore district, Karnataka, India. We considered similar dwelling habits, socioeconomic status, and geographical location for sampling. This was a field study conducted under the umbrella of the *M*ysuru st*u*dy on *D*eterminants of *H*ealth in *R*ural *A*dults (MUDHRA) [21–24].

## 2. Materials and Methods

### 2.1. Study Settings

Sampling for this field study was performed from eight randomly selected villages within the Nanjangud subdistrict, Mysore district, Karnataka, India, with similar socioeconomic and dwelling status. Due to gender-specific exposure [21, 22], only females were recruited for the BMS-exposed and only males for tobacco smoker subgroups (≥40 years of age). Out of the 8457 subjects initially screened for the MUDHRA cohort, there were 3953 women, of whom none had ever smoked [22]. Of the 4504 men screened initially, 2272 (50.44%) were smokers but none were exposed to biomass fuel smoke [23, 24].

### 2.2. Study Design and Subjects

The cohort characterization procedure and spirometry for this cross-sectional study has been previously described [21, 22]. Briefly, spirometry was carried out according to the American Thoracic Society (ATS) guidelines (ERS-ATS-COPD guidelines 2004). Postbronchodilator-forced expiratory volume in 1 second (FEV_1_) and forced vital capacity (FVC) were measured. COPD was diagnosed according to the global initiative for chronic obstructive lung disease (GOLD) criteria of persistent airflow obstruction determined by a postbronchodilator FEV_1_/FVC-ratio of <0.7. Disease severity of COPD was based on lung function according to GOLD guidelines.

Spirometry was performed using the EasyOne spirometer (ndd Medizintechnik; Zurich, Switzerland), and the subjects were requested to sit comfortably with loose clothing. Postbronchodilator testing (15 minutes after 200 micrograms of salbutamol *via* a metered dose inhaler and spacer) was performed. The postbronchodilator spirometry maneuvers had to satisfy ATS criteria for acceptability. It should have a minimum of three attempts, two of which should satisfy acceptability criteria (no artifacts, good start, sharp peak, no cough, no sudden, or premature stops) and the difference between the best and next best values of <200 ml for both FEV1 and FVC. The calibration of the spirometer was performed daily using a 3-liter syringe provided by the manufacturer. The reference values used were Asian (Chhabra).

A detailed validated questionnaire based on the Burden of Obstructive Lung Disease (BOLD) study translated in regional language (Kannada) was used to assess the exposure scenario and other background information. The field study was performed by trained field workers following a house-to-house visit. The field workers had earlier participated in the urban Mysore BOLD study and were trained in the BOLD protocol [21–23]. The questionnaire included demographic variables, various respiratory symptoms, and risk factors including tobacco smoking and biomass smoke exposure. All subjects were in stable condition and without any reported infection in the 4 weeks prior to blood sampling. Subjects with histories of any other respiratory disease like asthma and tuberculosis were excluded. Chest X-ray and sputum for acid-fast staining were performed in subjects with chest symptom to exclude tuberculosis. Subjects with any reported cardiovascular and metabolic diseases such as diabetes were also excluded. Most of the patients were naïve to inhaled corticosteroid/long-acting and beta-agonists (ICS/LABA) and did not take regular medications. Some of the participants had used medications for managing acute exacerbations.

The description of the study population is provided in Table 1. The study population constituted of 29 BMS-COPD **(**female), 24 BMS-exposed individuals without COPD (**BMS-CONTROL**, female), 23 TS-COPD (male), and 22 tobacco smokers without COPD (**TS-CONTROL**, male). Moderate and severe COPD patients constituted the majority (86–90%) of both BMS-COPD and TS-COPD groups (Table 1). BMS-COPD and BMS-CONTROL were matched for age, gender, and biomass exposure index. All subjects in the biomass smoke group were BMS-exposed. The TS-COPD subgroup was age- and gender-matched to TS-CONTROL but had slightly higher tobacco consumption (Table 1). All subjects in the tobacco smokers group were current smokers.

All procedures of this study (Indian cohort) were approved by Institutional Ethical Clearance JSS Medical College (JSSMC/IEC/13/4048/2016–2017), Mysore, Karnataka, India, according to the guidelines of the Indian Medical Research Council (ICMR). Informed and written consent was obtained from each subject participating in the study. It was not possible to recruit an adequate number of healthy subjects without biomass smoke and without tobacco smoke exposure **(TS-BMS-CONTROL)** from the same geographical region (Nanjangud subdistrict, Karnataka, India) with similar socioeconomic and dwelling status. Therefore, we used a Swedish cohort constituting of 15 subjects accessible to us as the TS-BMS-CONTROL subgroup (Table 1). All subjects in the Swedish cohort gave written informed consent, and the study was approved by the Ethics Committee, Stockholm, Sweden (protocol number 2010/2:8). For internal control experiments, we compared the serum chemokines and cytokines of Swedish TS-BMS-control (*n* = 15) with the Indian TS-BMS-control (*n* = 5). The median range of the 40 chemokines and cytokines was comparable among the two populations and showed no significant differences (data not shown).

### 2.3. Exposure Assessment

Biomass exposure index (**BMEI**) was calculated as previously described [22]. Briefly, the BMEI calculation was based on the average number of hours spent daily for cooking multiplied by the total number of years spent in cooking [25]. All the households used only dry firewood for their cooking and heating needs. None of the households in this study used dried dung or dried coconut shells for heating and cooking purposes. Tobacco smoke exposure is represented as the number of pack years.

### 2.4. Blood Collection

3 ml of venous blood sample was collected from each subject by trained professionals using the venipuncture method in the BD Vacutainer® PLUS plastic serum tubes with spray-coated silica. The tubes were incubated in an upright position at room temperature for 30 minutes and centrifuged for 15 minutes at 2500 RPM. The supernatant (serum) was carefully aspirated without disturbing the cell layer into prelabeled cryovials and stored at −80°C till further use.

### 2.5. Cytokine and Chemokine Panel Assay

Concentrations of forty cytokines and chemokines (Supplementary Table ST1) were measured using the Bio-Plex Pro™ Human Chemokine Panel assay kit (Bio-Rad, Cat#, 40-Plex 171AK99MR2) and Bio-Plex Multiplex immunoassay system (Bio-Rad Bio-Plex 200) according to the manufacturer's instruction. Bio-Plex Manager™ and Bio-Plex Data Pro™ Software were used to analyze the generated multiplex data. All the assays passed the quality control of the manufacturer. Data are represented as median (25th–75th percentile) pg/ml. All assays passed the quality control of the manufacturer. Out-of-range (<OOR) values were assigned the lowest detectable value. There were no >OOR values.

### 2.6. Statistics

Nonparametric Kruskal-Wallis followed by Mann-Whitney *U* tests, when appropriate, were performed to determine the statistical significance for the difference of cytokine and chemokine concentrations between the BMS-COPD, BMS-CONTROL, and TS-BMS-CONTROL or TS-COPD, TS-CONTROL, and TS-BMS-CONTROL groups. Spearman rank test was used to analyze the correlation between lung function, exposure, age, gender, and disease severity with chemokine and cytokine concentrations. Correlation was assessed only between significantly different analytes among BMS-COPD versus BMS-CONTROL groups and TS-COPD versus TS-CONTROL. *p* ≤ 0.01 was considered to be statistically significant. All statistical analyses were performed using GraphPad Prism software (Version: 5; La Jolla, California).

### 2.7. Protein-Protein Interaction Network Analysis

The web-based STRING tool (https://string-db.org/) [26] was used to assess the protein-protein network interaction between cytokines and chemokines exhibiting significantly different serum concentrations between (i) BMS-COPD versus BMS-CONTROL, (ii) TS-COPD versus TS-CONTROL, (iii) TS-COPD versus BMS-COPD, and (iv) TS-CONTROL and BMS-CONTROL groups.

## 3. Results

BMS-COPD and BMS-CONTROL subgroups were matched for age, sex (female), and biomass smoke exposure levels. The tobacco exposure was slightly higher among the TS-COPD subgroup compared to the TS-CONTROL subgroup (Table 1). The TS-COPD and TS-CONTROL groups were matched for age and sex (male). Due to gender-specific exposure to biomass smoke (women) and tobacco smoke (men) in rural India, the biomass exposure groups and tobacco smokers group could not be gender-matched.

### 3.1. Cytokine and Chemokine Profiles among Different Subgroups

Serum concentrations of three cytokines (Table 2) were significantly higher in BMS-COPD compared to BMS-CONTROL subjects. On the other hand, serum concentrations of nine cytokines were significantly different between TS-COPD and TS-CONTROL groups (Table 2). Comparisons of chemokine and cytokine signatures between the different subgroups to answer the following questions are detailed below.

**Figure 1:**
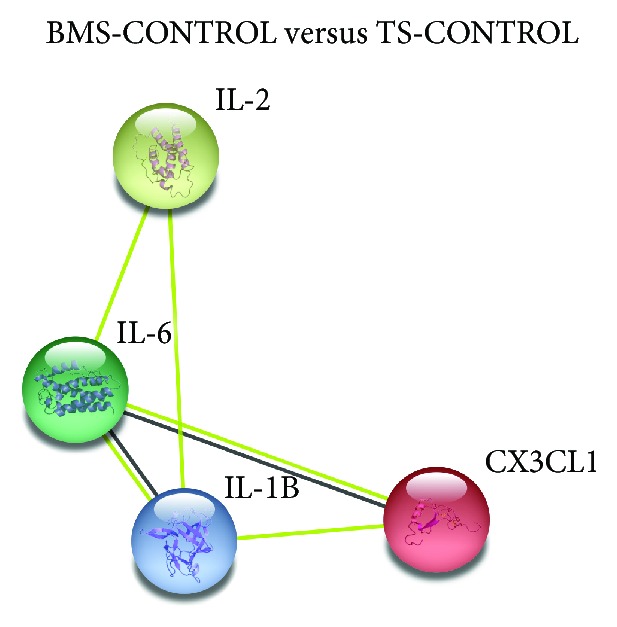
Protein-protein interaction of chemokines and cytokines exhibiting significantly (*p* ≤ 0.01) altered serum concentrations among biomass-exposed subjects without COPD (BMS-CONTROL) versus tobacco smokers without COPD (TS-CONTROL). CX3CL: C-X3-C motif chemokine ligand; IL: interleukin.

*(i) BMS-CONTROL vs TS-CONTROL vs TS-BMS-CONTROL*. Does biomass smoke exposure or smoking lead to alterations in chemokine and cytokine signature, and is there a difference between biomass exposure and smoking? (Figure 1 and Supplementary Table ST2)

**Figure 2:**
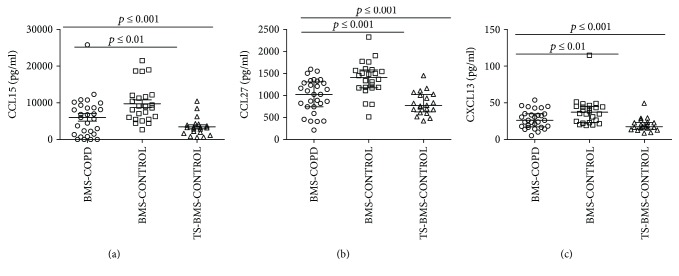
Increased serum concentrations of chemokine: (a) C-C motif ligand 15 (CCL15), (b) CCL27, and (c) C-X-C motif chemokine ligand 13 (CXCL13) was detected among biomass smoke-exposed subjects with chronic obstructive pulmonary disease (BMS-COPD) compared to biomass smoke-exposed subjects without COPD (BMS-CONTROL). Data are represented as median (25th–75th percentile) pg/ml. Statistical analysis was performed using the nonparametric Kruskal-Wallis followed by Mann-Whitney tests, when appropriate. *p* ≤ 0.01 was considered as statistically significant. COPD: chronic obstructive pulmonary disease; TS-BMS-CONTROL: no tobacco- and no biomass smoke-exposed subjects.

*(ii) BMS-COPD vs BMS-CONTROL vs TS-BMS-CONTROL*. Is COPD associated with alterations to chemokine and cytokine signature in biomass smoke exposure? (Figure 2 and Table 2)

**Figure 3:**
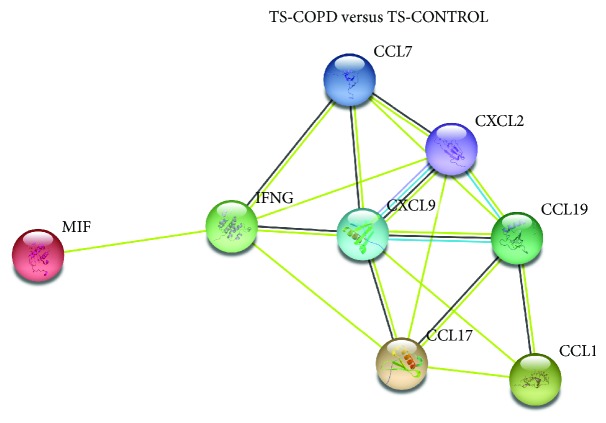
Protein-protein interaction of chemokines and cytokines exhibiting significantly (*p* ≤ 0.01) altered serum concentrations among (a) tobacco smokers with COPD (TS-COPD) versus tobacco smokers without COPD (TS-CONTROL). CCL: chemokine (C-C motif) ligand; CXCL: C-X-C motif chemokine ligand IFNG: interferon gamma; IL: interleukin; MIF: macrophage migration inhibitory factor.

*(iii) TS-COPD vs TS-CONTROL vs TS-BMS-CONTROL*. Is COPD associated with alterations to chemokine and cytokine signature in smokers? (Figure 3 and Table 2)

**Figure 4:**
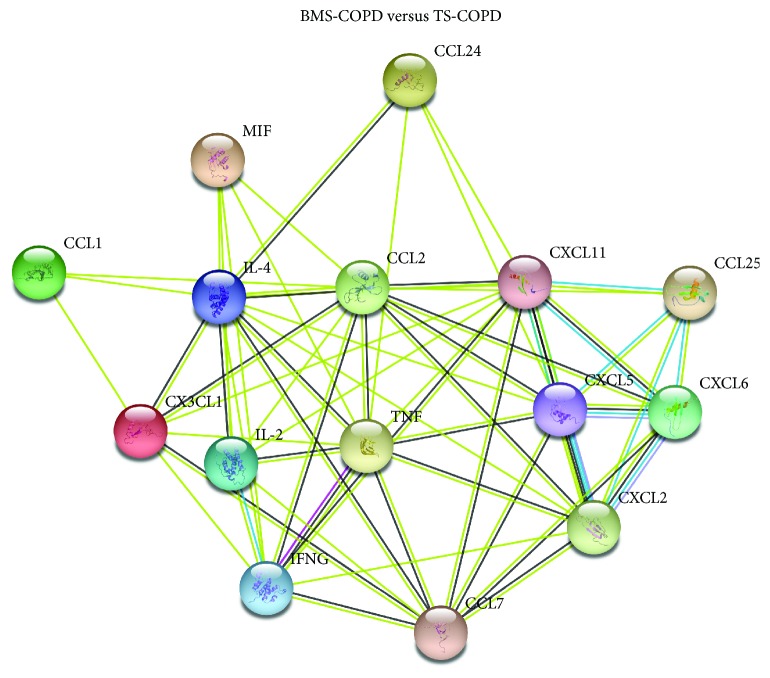
Protein-protein interaction of chemokines and cytokines exhibiting significantly (*p* ≤ 0.01) altered serum concentrations among (a) tobacco smokers with COPD (TS-COPD) versus biomass exposed subjects with COPD (BMS-COPD). CCL: chemokine (C-C motif) ligand; CX3CL: C-X3-C motif chemokine ligand; CXCL: C-X-C motif chemokine ligand; IFNG: interferon gamma; IL: interleukin; MIF: macrophage migration inhibitory factor; TNF: tumor necrosis factor alpha.

*(iv) BMS-COPD vs TS-COPD vs TS-BMS-CONTROL*. Is there a difference in chemokine and cytokine signature between COPD induced by biomass smoke exposure and smoking? (Figure 4 and Supplementary Table ST2)

*(i) TS-CONTROL versus BMS-CONTROL versus TS-BMS-CONTROL*. Concentrations of 4 analytes (3 higher and 1 lower) were significantly different in BMS-CONTROL compared to TS-CONTROL subjects. The cytokines and chemokines include CX3CL1, IL-1B, 2, and 6 (Supplementary Table ST2). IL-1B exhibited lower concentrations in BMS-CONTROL compared to TS-CONTROL subjects whereas CX3CL1, IL-2, and IL-6 were higher. A protein-protein interaction of these 4 analytes is shown in Figure 1.

*(ii) BMS-COPD versus BMS-CONTROL versus TS-BMS-CONTROL*. Significantly higher levels of 3 chemokines [chemokine (C-C motif) ligand- (CCL-) 15 and 27 and C-X-C motif chemokine ligand- (CXCL-) 13] were detected in BMS-CONTROL compared to the BMS-COPD group (Figures 2(a)–2(c)). Protein-protein interaction studies revealed a close interaction among CCL27 and CXCL13 but not CCL15. A summary of serum concentrations is provided in Table 2. No correlation was detected between serum concentrations of CCL15, CCL27, and CXCL13 with lung function and/or COPD severity (Supplementary Table ST3).

*(iii) TS-COPD versus TS-CONTROL versus TS-BMS-CONTROL*. Higher serum concentrations of nine analytes [CCL-1, 7, 15, 17, and 19; CXCL-2 and 9, interferon gamma (IFNG), and macrophage migration inhibitory factor (MIF)] were detected in the TS-CONTROL group compared to the TS-COPD group. A summary of serum concentrations is presented in Table 2. Weak correlations with lung function and/or COPD severity with the concentrations of some of the chemokines and cytokine have been detected (Supplementary Table ST3). A protein-protein network analysis of the chemokines and cytokines exhibiting significantly higher serum concentrations in TS-CONTROL compared to TS-COPD is presented in Figure 3.

*(iv) TS-COPD versus BMS-COPD versus TS-BMS-CONTROL*. Concentrations of 15 analytes (14 higher and 1 lower) were significantly different in BMS-COPD compared to TS-COPD subjects. The cytokines and chemokines include CCL-1, 2, 7, 24, and 25; C-X-C motif 3 chemokine ligand 1 (CX3CL1); CXCL-2, 5, 6, and 11; IFN-G; IL-2 and 4; MIF; and tumor necrosis factor alpha (TNF-A) (Supplementary Table ST2). CXCL11 exhibited lower concentrations in BMS-COPD compared to TS-COPD subjects. A protein-protein interaction of these 15 analytes is shown in Figure 4.

Correlation between the concentration of the chemokines and cytokines with age, gender, tobacco load, and BMEI was not detected.

## 4. Discussion

Findings of this study indicate an exposure-specific (biomass and tobacco smoke) systemic chemokine and cytokine signature among subjects with and without COPD within the investigated rural south Indian population. Based on the protein-protein interaction profile, it is suggestive that the serum chemokines and cytokines exhibiting higher levels (CCL-1, 7, 15, 17, and 19; CXCL-2 and 9; IFNG; and MIF) in the TS-CONTROL compared to the TS-COPD group present a distinct molecular signature (Figure 3). *In silico* analysis did not exhibit any interaction of CCL15 within this cascade and therefore is not included in the protein-protein network. In case of biomass smoke exposure, higher levels of CCL15, CCL27, and CXCL13 were detected in the BMS-CONTROL group compared to the BMS-COPD subgroup. Higher levels of several chemokines and cytokines among the TS-CONTROL and BMS-CONTROL groups compared to their respective COPD subgroup is indicative of their pleiotropic functions. Therefore, it is important to understand not only the pathogenic role of the chemokines and cytokines in COPD but also their protective/homeostatic action.

Systemic inflammation is an integral event of COPD although the lung is the actual disease site. It has also been often observed that the systemic chemokine and cytokine profiles do not complement the local (lung) molecular signature. The various proposed theories which exist to explain the systemic inflammation in COPD are as follows [18, 27–29]: (i) spillover of airway and lung parenchyma inflammation into the systemic circulation, (ii) tobacco smoke-driven systemic inflammation as in the case of cardiovascular diseases, (iii) lung pathophysiological change (hyperinflation and hypoxia) driven systemic inflammation, (iv) low-grade systemic inflammation during the normal ageing process, and (v) production of systemic inflammatory mediators into the blood from other body parts like skeletal muscle and bone marrow. This led us to screen a comprehensive panel of forty chemokines and cytokines in our pilot study to identify extrinsic exposure-specific systemic molecular profiles within the biomass smoke- and tobacco smoke-exposed subgroups. Interestingly, CCL27 and CXCL13, which are increased in BMS-CONTROL compared to BMS-COPD, are classified as homeostatic chemokines [30]. Previously, we identified an activation of a homeostatic/defense response reaction while studying a panel of chemokines and cytokines in the lungs of mouse to overcome carbon nanoparticle challenge [31].

The chemokine system controls the immune cell migration and positioning at the organismic level during homeostasis, acute inflammation, and regulation of immune responses [32]. The fine balance of chemokines during inflammatory process, immune cell trafficking, and homeostasis is critical between health and disease states [33]. Based on the expression pattern and function, chemokines are grouped as inflammatory and homeostatic (leukocyte mobilizing during inflammation) [33]. Inflammatory chemokines are defined as those which are upregulated during inflammation and are mainly involved in the process of recruitment of leukocytes to the inflamed tissue [30]. Homeostatic cytokines on the other hand are expressed constitutively in lymphoid and other organs and mediate normal physiological migration and homing of various cells including lymphocytes [30]. Some chemokines on the other hand exhibit overlap of both inflammatory and homeostatic functions and are therefore referred to as dual-function chemokines [30].

Based on the functional categorization of chemokines as described by Zlotnik and Yoshie [30], the elevated chemokines in TS-CONTROL compared to TS-COPD can be classified as follows: Inflammatory (CCL1, 7, and 15; CXCL2 and 9), homeostatic (CCL19, CCL25), and dual function (CCL17). An increased level of IFN-G is associated with decreased number of Th2 cells, enhancement of COPD severity, and decreased asthma [34]. However, we detected a moderate negative correlation between IFN-G concentrations and COPD severity in our study population (Supplementary Table ST3). Higher serum MIF levels have been reported in patients with COPD compared with controls. Increased MIF levels have been also observed during acute exacerbations of COPD [35]. Though not significant, the authors reported a decreasing trend of MIF levels in higher COPD-GOLD categories in this study [35]. We detected a moderate negative correlation with MIF concentrations and COPD severity in our study population (Supplementary Table ST3). It would be important in future course of studies to understand the interdependence among these chemokines and cytokines to understand the complex molecular cascade during COPD onset and disease progression.

Analysis of the serum cytokine and chemokine panel in the biomass-exposed group revealed higher levels of CCL15 (inflammatory), CCL27 (homeostatic), and CXCL13 (homeostatic) in BMS-CONTROL subjects compared to BMS-COPD. CCL15 or leukotactin-1 is a chemoattractant for T cells and monocytes and acts through C-C chemokine receptor type 1 (CCR1). Increased levels of transcript and circulating levels of CCL15 have been associated with atherosclerosis. *In vitro* studies demonstrated increased production of CCL15 in response to oxidative stress in macrophages, macrophage-derived foamy cells, and endothelial cells [36]. Airway smooth muscle cells are a potent source of CCL15 in the lung. Increased levels of CCL15 in the lung have been associated with airway inflammation [37]. Interestingly, CCL15 is the only common analyte increased in both TS-CONTROL and BMS-CONTROL compared to TS-COPD and BMS-COPD, respectively. The elevated levels of CCL27 and CXCL13 appear to be involved in a homeostatic axis that distinguishes the COPD and non-COPD subjects following long-term biomass exposure. CCL27 plays an important role in the immune-inflammatory processes in many skin diseases [38]. It is constitutively produced by epidermal keratinocytes and participates in tissue-specific homing of lymphocytes [39]. Increased level of systemic CCL27 among stable COPD patients (no exacerbation during previous 4 weeks) compared with controls [40] is suggestive of a homeostatic response [41]. Sources of CXCL13 in the lung are the follicular dendritic cells in healthy conditions. B cells also produce CXCL13 in COPD lungs [41] and have been involved in lymphoid neogenesis [42]. In COPD, CXCL13 promotes recruitment of B cells into lymphoid follicles and their compartmentalization within lymphoid follicles [41]. Increased circulatory CXCL13 levels have been associated with atherosclerotic plaque stabilization indicating its plausible homeostatic function [43].

## 5. Strengths and Limitations

One of the strengths of this study is the biomass exposure and age- and gender-matched BMS-COPD and BMS-CONTROL subgroups. However, biomass smoke exposure assessment in this study was performed using the traditional questionnaire-based survey. Even though study subgroups were nonoverlapping in nature (i.e., individuals exposed to biomass smoke were not tobacco smokers and *vice versa*), it would have been more appropriate to measure the actual biomass smoke exposure through the assessment of CO, PM_10_, PM_2.5_, etc. Lack of actual exposure measurement may mis-classify exposure severity. The tobacco consumption was slightly higher among TS-COPD subgroups compared to TS-CONTROL, but the two subgroups were age- and gender-matched. Comparison of a comprehensive panel of 40 interacting chemokines and cytokines among the various subgroups provides a broad screening range for plausible systemic biomarkers that may play a role in COPD pathogenesis and/or resistance among individuals with comparable exposure levels. However, the findings are limited by the relatively small sample size and cross-sectional design of the study. Lack of adequate number of TS-BMS-CONTROL subjects from the same geographical region in India may influence the findings. Moreover, the TS-BMS-CONTROL used in this study from Swedish origin is quite different from the biomass smoke-exposed subjects as the latter is linked to poverty and many associated health risk factors. However, as discussed earlier, we did not detect any significant differences in the serum concentrations of the 40 chemokines and cytokines analyzed between the Swedish and Indian TS-BMS-CONTROL subjects. Due to the gender-specific exposure scenario, the biomass exposed- and tobacco smokers' subgroups were limited to women and men, respectively. We are also unaware of the childhood biomass smoke exposure levels among these individuals, as children are often indoors with their mothers during cooking hours which may result in impaired lung function development in early life.

## 6. Conclusions

To summarize, findings of the current study indicate a plausible exposure-specific patho-physiological profile for tobacco smoke- and biomass smoke-induced COPD. To our knowledge, this is one of the few studies comparing groups with matched exposure load, age, sex, dwelling conditions, and socioeconomic status to elucidate systemic chemokine and cytokine signature using a comprehensive panel of markers for biomass smoke-induced COPD. CCL27 and CXCL13 may be considered as putative homeostatic/protective biomarkers for biomass-induced COPD within the investigated South Indian population. The findings of the study warrant validation in larger and multiple cohorts.

## Figures and Tables

**Table 1:** Characteristics of the study cohort. All data are presented as median (25th–75th percentile).

	BMS-COPD^#^^∗^	BMS-CONTROL^∗^	TS-COPD^#$^	TS-CONTROL^$^	TS-BMS-CONTROL
Number of subjects	29	24	23	22	15
Gender (male/female)	0/29	0/24	23/0	22/0	6/9
Age in years (mean, range)	60.31 (44–78)	61.63 (43–83)	63.65 (48–78)	59.27 (40–75)	60.47 (46–71)
FEV_1_ (% of predicted) (mean ± SE)	51.48 ± 2.60	104.41 ± 2.87	57.61 ± 3.22	106.41 ± 3.09	95.47 ± 3.68
FEV_1_/FVC (mean ± SE)	0.61 ± 0.01	0.82 ± 0.008	0.61 ± 0.01	0.83 ± 0.01	0.78 ± 0.02
Pack years/biomass exposure index (BMEI) [median (25th–75th percentile)]	112 (88–132)	120 (84.75–151)	22.2 (15.85–30)^∗^ ^∗^ *p* = 0.03 compared to TS-CONTROL	16.15 (12.15–18.15)	—

^#^BMS-COPD: mild 1, moderate: 16, severe: 10, very severe: 2; TS-COPD: mild 2, moderate: 15, severe: 5, very severe: 1. ^∗^Current BMS exposed; ^$^current tobacco smokers. COPD: chronic obstructive pulmonary disease; TS-COPD: tobacco smokers with COPD; TS-CONTROL: tobacco smokers without COPD; BMS-COPD: biomass smoke exposed subjects with COPD; BMS-CONTROL: biomass exposed subjects without COPD; TS-BMS-CONTROL: no tobacco- and no biomass smoke exposed subjects; FEV_1_: forced expiratory volume in 1 second (postbronchodilator challenge); FVC: forced vital capacity.

**Table 2:** Summarized representation of the significantly different serum cytokine and chemokine concentrations [median (25th–75th percentile) pg/ml] between TS-COPD versus TS-CONTROL and BMS-COPD versus BMS-CONTROL groups. TS-CONTROL: tobacco smokers without COPD; BMS CONTROL: biomass smoke exposed without COPD; TS-BMS-CONTROL: no tobacco- and no biomass smoke-exposed subjects; *p* ≤ 0.01 was considered as statistically significant.

	(pg/ml)	(pg/ml)	(pg/ml)	*p* value	*p* value
	TS-COPD	TS-CONTROL	TS-BMS-CONTROL	Kruskal-Wallis	Mann-Whitney *U*
CCL1	27.38 (24.26–44.77)	50.87 (38.54–54.26)	23.44 (25.95–22.26)	≤0.001	≤0.001
CCL7	22.18 (13.32–38.00)	49.66 (27.315–55.81)	23.99 (28.02–22.79)	≤0.01	≤0.01
CCL15	2979.58 (1016.3–4740.20)	8287.66 (5566.82–10180.96)	3270.56 (4118.67–2526.68)	≤0.01	≤0.001
CCL17	69.05 (39.80–155.46)	168.52 (111.65–205.07)	155.01 (224.51–147.55)	≤0.01	≤0.01
CCL19	124.46 (58.29–196.73)	204.90 (155.81–272.63)	252.12 (281.37–211.52)	≤0.01	≤0.01
CXCL2	268.42 (187.28–397.49)	753.46 (290.37–857.49)	199.75 (281.82–172.32)	≤0.01	≤0.01
CXCL9	356.22 (239.24–518.25)	601.80 (524.54–701.57)	256.71 (277.51–227.55)	≤0.01	≤0.01
IFN-G	1.43 (1.05–35.63)	47.92 (28.52–59.58)	1.31 (1.42–1.21)	≤0.001	≤0.01
MIF	2063.61 (724.04–4329.13)	14115.77 (2950.2–17033.25)	1344.22 (1825.87–564.61)	≤0.001	≤0.01
	BMS-COPD	BMS-CONTROL	TS-BMS-CONTROL	Kruskal-Wallis	Mann-Whitney *U*
CCL15	5977.22 (1459.25–8646.12)	8799.35 (5885.19–11715.51)	3270.56 (4118.67–2526.68)	≤0.001	≤0.01
CCL27	1024.99 (743.09–1285.01)	1409.14 (1179.38–1569.95)	593.96 (516.40–1028.81)	≤0.001	≤0.001
CXCL13	26.03 (17.92–34.85)	37.14 (24.24–44.64)	22.77 (19.62–29.48)	≤0.001	≤0.01

## Data Availability

All data required to comprehend the manuscript have been provided in the manuscript main body and supplementary material. All raw data are available with the corresponding authors.

## Abbreviations

BMS-COPD: Biomass smoke-exposed subjects with chronic obstructive pulmonary disease
BMS-CONTROL: Biomass-exposed subjects without COPD
CCL: Chemokine (C-C motif) ligand
COPD: Chronic obstructive pulmonary disease
CX3CL: C-X3-C motif chemokine ligand
CXCL: C-X-C motif chemokine ligand
FEV_1_: Forced expiratory volume 1 second (postbronchodilator challenge)
FVC: Forced vital capacity
IFNG: Interferon gamma
IL: Interleukin
MIF: Macrophage migration inhibitory factor
TNF: Tumor necrosis factor alpha
TS-CONTROL: Tobacco smokers without COPD
TS-COPD: Tobacco smokers with COPD
TS-BMS-CONTROL: No tobacco- and no biomass smoke-exposed subjects.

## Supplementary Materials

Supplementary Materials Supplementary Table ST1: serum cytokine and chemokine concentrations in the study subjects. *n* = number of subjects with detectable analyte concentration in each group. Data are represented as median (25th–75th percentile) pg/ml. Supplementary Table ST2: summarized representation of the significantly different serum chemokines and cytokines between tobacco smokers with chronic obstructive pulmonary disease (TS-COPD) versus biomass smoke-exposed subjects with COPD (BMS-COPD) and TS-CONTROL versus BMS-CONTROL groups. Supplementary Table ST3: summarized representation of the significantly different serum chemokine and cytokine concentrations between TS-COPD versus TS-CONTROL and BMS-COPD versus BMS-CONTROL groups and their correlation to lung function and COPD severity.
