# Supplementary material for: Putative Systemic Biomarkers of Biomass Smoke-Induced Chronic Obstructive Pulmonary Disease among Women in a Rural South Indian Population
Source: Dis Markers. 2018 Nov 22;2018:4949175. doi: 10.1155/2018/4949175 (PMC6282129; doi:10.1155/2018/4949175)
Supplement: Supplementary Materials — Supplementary Table ST1: serum cytokine and chemokine concentrations in the study subjects. n = number of subjects with detectable analyte concentration in each group. Data are represented as median (25th–75th percentile) pg/ml. Supplementary Table ST2: summarized representation of the significantly different serum chemokines and cytokines between tobacco smokers with chronic obstructive pulmonary disease (TS-COPD) versus biomass smoke-exposed subjects with COPD (BMS-COPD) and TS-CONTROL versus BMS-CONTROL groups. Supplementary Table ST3: summarized representation of the significantly different serum chemokine and cytokine concentrations between TS-COPD versus TS-CONTROL and BMS-COPD versus BMS-CONTROL groups and their correlation to lung function and COPD severity. [file 4949175.f1.docx]

**Additional file**

**Putative systemic biomarkers of biomass smoke induced chronic obstructive pulmonary disease among women in a rural south Indian population**

Sangeetha Vishweswaraiah^1*^, Tania Ahalya Thimraj^1*^, Leema George^1^, Chaya Sindaghatta Krishnarao^2^, Komarla Sundararaja Lokesh^2^, Jayaraj Biligere Siddaiah^2^, Kjell Larsson^3^, Swapna Upadhyay^3^, Lena Palmberg^3^, Mahesh Padukudru Anand^2#^, Koustav Ganguly^1,3#^

^1^SRM Research Institute, SRM Institute of Science and Technology (Formerly SRM University), Chennai, 603203, India

^2^JSS Medical College and Hospital, JSS Academy of Higher Education and Research, Department of Pulmonary Medicine, Mysuru, India

^3^Work Environment Toxicology; Institute of Environmental Medicine, Karolinska Institutet; Box 287, SE-171 77 Stockholm, Sweden

**^*^ Contributed equally;**

**^*#^ Contributed equally and corresponding authors**

| **Name** | **Email** |
| --- | --- |
| Sangeetha Vishweswaraiah | sangeethav.srm@gmail.com |
| Tania A Thimraj | taniathimraj.srm@gmail.com |
| Leema George | leemag.srm@gmail.com |
| Chaya SK | chaya.sindaghatta@gmail.com |
| Lokesh KS | kslokesh@gmail.com |
| Jayaraj BS | drjayarajbs@yahoo.com |
| Kjell Larsson | Kjell.larsson@ki.se |
| Swapna Upadhyay | swapna.upadhyay@ki.se |
| Lena Palmberg | lena.palmberg@ki.se |
| Mahesh PA | mahesh1971in@yahoo.com |
| Koustav Ganguly | koustav.ganguly@ki.se |

**Correspondence:**

**Koustav Ganguly**

Work Environment Toxicology; Institute of Environmental Medicine, Karolinska Institutet; Box
287, SE-171 77 Stockholm, Sweden; **Email:** koustav.ganguly@ki.se; Phone: +46-707646787

**Mahesh PA**

Department of Pulmonary Medicine, JSS Medical College and Hospital, JSS Academy of Higher Education and Research, Shivarathreeshwara Nagar, Mysuru, 570015, **Email:** mahesh1971in@yahoo.com; India. Phone: +91-9448044003

**Conflict of Interest:** None

This study was supported by DBT-India**,** VINNOVA and Swedish Heart Lung Foundation.

**Supplementary table ST1:** Serum cytokine and chemokine concentrations in the study subjects. n= number of subjects with detectable analyte concentration in each group. Data are represented as median (25^th^ - 75^th^ percentile) pg/ml

**Abbreviations: TS-COPD:** Tobacco smokers with COPD; **TS-CONTROL:** Tobacco smokers without COPD; **BMS-COPD:** Biomass smoke exposed subjects with COPD; **BMS-CONTROL:** Biomass exposed subjects without COPD; **TS-BMS-CONTROL:** No Tobacco- and no biomass smoke exposed subjects **CCL:** Chemokine (C-C motif) ligand **CX3CL1:** C-X-C Motif 3 Chemokine Ligand 1; **CXCL:** C-X-C Motif Chemokine Ligand **GM-CSF:** Granulocyte-macrophage colony-stimulating factor; **IFNG:** Interferon gamma; **IL:** Interleukin; **MIF:** Macrophage migration inhibitory factor; **TNF-A**: Tumor necrosis factor alpha.

| **Cytokine** | **TS-COPD**  **(n=23)** | **TS-CONTROL**  **(n=22)** | **BMS-COPD**  **(n=29)** | **BMS-CONTROL**  **(n=24)** | **TS-BMS-CONTROL (n=15)** |
| --- | --- | --- | --- | --- | --- |
| **CCL1** | 27.38 (24.26-44.77) | 50.87 (38.54-54.26) | 52.07 (47.91-55.33) | 54.60 (49.98-60.11) | 23.44 (22.25-25.95) |
| **CCL2** | 23.29 (16.44-32.39) | 27.14 (23.90-31.50) | 35.14 (31.75- 43.09) | 31.90 (25.80-38.36) | 57.61 (43.82-66.77) |
| **CCL3** | 10.71 (5.74-14.86) | 9.01 (6.57-11.12) | 9.21 (6.45-14.78) | 8.31 (7.02-9.93) | 5.61 (4.61-6.62) |
| **CCL7** | 22.18 (13.32-38.01) | 49.67 (27.32- 55.81) | 54.86 (45.67-63.59) | 54.85 (48.35-67.51) | 23.99 (22.79-28.02) |
| **CCL8** | 40.05 (19.34-67.39) | 61.72 (37.73-80.68) | 36.52 (31.62-60.96) | 39.84 (33.92- 44.45) | 48.85 (42.5-52.19) |
| **CCL11** | 76.24 (51.03- 108.37) | 66.67 (58.46-71.72) | 59.82 (49.39-74.48) | 68.45 (52.32-72.61) | 122.27(98.36-146.56) |
| **CCL13** | 63.28 (40.16- 111.48) | 98.83 (81.81-111.88) | 93.97 (61.04-127.75) | 82.04 (62.54-101.3) | 96.45 (84.81-107.415) |
| **CCL15** | 2979.58(1016.3-4740.21) | 8287.66 (5566.82- 10180.96) | 5977.22 (1459.25-8646.12) | 8799.35 (5885.19- 11715.51) | 3270.56 (2526.69-4118.67) |
| **CCL17** | 69.05 (39.80-155.46) | 168.52 (111.65-205.07) | 146.51 (100.24- 195.99) | 137.37 (116.46- 303.80) | 155.01 (147.55-224.51) |
| **CCL19** | 124.46 (58.30-196.74) | 204.90 (155.81- 272.63) | 195.98 (157.06- 249.15) | 197.01 (163.75- 383.16) | 252.12(211.52-28.38) |
| **Cytokine** | **TS-COPD** | **TS-CONTROL** | **BMS-COPD** | **BMS-CONTROL** | **TS-BMS-CONTROL** |
| **CCL20** | 9.97 (6.59- 15.53) | 16.89 (12.07- 23.64) | 17.52 (8.6- 20.54) | 20.64 (16.04- 25.08) | 5.54(4.61-6.04) |
| **CCL21** | 1890.92(1582.94-2705.39) | 2195.91 (1666.72- 2609.78) | 2042.55 (1655.03- 2569.38) | 2491.05 (1619.32- 2781.32) | 1992.86 (1617.84-2261.56) |
| **CCL22** | 383.12(236.3-645.64) | 720.98 (344.08- 851.95) | 566.17 (438.86- 784.15) | 656.56 (575.27- 761.05) | 549.17(429.41-601.69) |
| **CCL23** | 305.48 (210.62- 468.59) | 325.3 (254.81- 347.41) | 333.1 (265.56- 381.66) | 343.34 (303.9- 380.15) | 269.48 (181.13-295.08) |
| **CCL24** | 176.85 (115.66- 518.415) | 500.28 (278.43- 566.70) | 661.26 (357.91- 1003.77) | 528.73 (421.46- 692.83) | 207.12 (124.88-281.74) |
| **CCL25** | 380.41(283.72-432.99) | 487.35 (384.82- 609.93) | 558.81 (421.87- 662.76) | 542.34 (494.23- 689.13) | 316.77 (270.06-345.53) |
| **CCL26** | 5.03 (4.55- 9.01) | 6.66 (5.61- 8.65) | 7.66 (5.16-9.46) | 8.315 (6.59- 9.07) | 9.08(6.24-10.44) |
| **CCL27** | 928.31 (667.62- 1162.41) | 1230.33 (782.88- 1502.47) | 1024.99 (743.09- 1285.01) | 1409.14 (1179.38- 1569.95) | 775.56 (688.32-972.54) |
| **CX3CL1** | 231.98 (164.365- 411.28) | 355.36 (243.79- 397.98) | 407.02 (366.85- 576.72) | 534.84 (392.46- 634.85) | 158.38(118.56-174.68) |
| **CXCL1** | 140.47 (118.80- 277.31) | 254.04 (186.91- 294.37) | 239.77 (201.27- 327.36) | 239.17 (202.97- 283.26) | 126.37(120.41-156.47) |
| **CXCL2** | 268.42 (187.28- 397.49) | 753.47 (290.38- 857.50) | 417.89 (272.62- 559.7) | 347.89 (295.72- 540.51) | 199.75(281.83-172.33) |
| **CXCL5** | 311.27(98.06-741.31) | 783.48 (1163.51-426.4) | 1426.93 (729.78- 2140.41) | 1028.50 (736.48- 1472.09) | 575.00(332.67-990.96) |
| **CXCL6** | 42.39 (26.37- 57.02) | 62.02 (42- 67.33) | 61.23 (51.71- 80.76) | 54.32 (40.34- 69.66) | 39.23(28.9-54.415) |
| **CXCL9** | 356.22 (239.24- 518.25) | 601.8 (524.54- 701.56) | 484.78 (375.36- 689.19) | 508.25 (421.22- 730.65) | 256.71(227.55-277.51) |
| **CXCL10** | 91.55 (37.53- 177.17) | 297.36(109.75-365.21) | 133.98 (76.16- 224.97) | 230.54 (148.86- 288.28) | 42.04(34.49-55.63) |
| **Cytokine** | **TS-COPD** | **TS-CONTROL** | **BMS-COPD** | **BMS-CONTROL** | **TS-BMS-CONTROL** |
| **CXCL11** | 145.75(31.21-402.13) | 61.07 (47.36- 75.20) | 46.74 (25.99- 67.04) | 54.27 (40.3- 66.08) | 189.05 (160.16-213.18) |
| **CXCL12** | 659.64 (513.46- 885.92) | 729.7 (524.4- 819.71) | 591.91 (431.81- 772.46) | 551.80 (464.45- 786.11) | 931.03(723.13-1104.08) |
| **CXCL13** | 31.02 (15.64- 55.50) | 33.67 (27.24- 43.82) | 26.03 (17.92- 34.85) | 37.14 (24.23- 44.64) | 16.38(13.13-21.36) |
| **CXCL16** | 367.67 (275.23- 564.61) | 487.41 (304.69- 551.23) | 498.14 (369.53- 607.11) | 571.45 (412.07- 622.12) | 246.07 (201.70-314.75) |
| **GM-CSF** | Not detectable | Not detectable | Not detectable | Not detectable | Not detectable |
| **IFNG** | 1.43 (1.05- 35.63) | 47.92 (28.52- 59.58) | 49.75 (42.35- 62.2) | 55.15 (45.15- 66.09) | 1.31(1.22-1.42) |
| **IL-1B** | 5.99 (2.79- 7.51) | 6.50 (3.68- 11.77) | 2.34 (2.03- 5.10) | 2.85 (2.32- 3.62) | 6.8(6.18-8.18) |
| **IL-2** | 4.4 (2.95- 10.09) | 12.3 (5.82- 15.30) | 14.83 (12.3- 17.97) | 15.78 (13.41- 20.24) | 4.17(3.84-4.73) |
| **IL-4** | 7.37 (5.87- 26.53) | 27.83 (24.15- 30.49) | 26.53 (22.35- 29.72) | 28.79 (27.83- 30.94) | 6.47(5.82-7.33) |
| **IL-6** | 16.92 (6.26- 46.84) | 12.59 (10.22- 13.81) | 15.4 (13.27- 17.22) | 16.01 (12.59- 22.1) | 9.63(8.52-10.17) |
| **IL-8** | 24.59 (14.33- 72.93) | 21.12 (12- 27.85) | 25.94 (18.92- 45.83) | 24.19 (20.52- 30.31) | 17.28(12.51-18.51) |
| **IL-10** | 22.365 (12.71- 29.65) | 27.26 (23.10- 31.81) | 27.79 (23.23- 30.49) | 26.76 (25.34- 38.32) | 36.64(31.48-40.8) |
| **IL-16** | 329.73 (232.87- 543.43) | 846.38 (397.30- 1282.01) | 555.57 (358.15- 949.65) | 842.70 (402.66- 1060.21) | 156.08 (170.15-129.74) |
| **MIF** | 2489.02 (862.48- 4423.36) | 14115.77 (2950.2- 17033.25) | 12089.63 (3528.79- 21472.19) | 15392.62 (6134.61- 18643.275) | 1344.22(564.61- 1825.88) |
| **TNF-A** | 13.2 (11.50- 19.16) | 21.12 (16.59- 24.58) | 21.44 (16.28- 27.36) | 22.16 (21.04-24.50) | 11.15(10.20-12.65) |

**Supplementary table ST2:** Summarized representation of the significantly different serum chemokines and cytokines between tobacco smokers with chronic obstructive pulmonary disease (TS-COPD) versus biomass smoke exposed subjects with COPD (BMS-COPD) and TS-CONTROL versus BMS-CONTROL groups.

**Abbreviations: TS-COPD:** Tobacco smokers with COPD; **TS-CONTROL:** Tobacco smokers without COPD; **BMS-COPD:** Biomass smoke exposed subjects with COPD; **BMS-CONTROL:** Biomass smoke exposed without COPD; **TS-BMS-CONTROL:** No Tobacco smoke no biomass exposed subjects;

Concentrations are represented as [median (25^th^-75^th^ percentile] pg/ml. p≤0.01 was considered as statistically significant.

| **Cytokine** | **TS-COPD**  **(pg/ml)** | **BMS-COPD**  **(pg/ml)** | | | **TS-BMS-CONTROL**  **(pg/ml)** | | **Kruskal-Wallis**  **p value** | | **Mann-Whitney**  **[TS-COPD**  **Versus**  **BMS-COPD]**  **p value** |
| --- | --- | --- | --- | --- | --- | --- | --- | --- | --- |
| **CCL1** | 27.38 (24.26-44.77) | 52.07 (47.91-55.33) | | | 23.44 (22.25-25.95) | | ≤0.001 | | ≤0.001 |
| **CCL2** | 23.29 (16.44-32.39) | 35.14 (31.75- 43.09) | | | 57.61 (43.82-66.77) | | ≤0.001 | | ≤0.01 |
| **CCL7** | 22.18 (13.32-38.01) | 56.76 (45.67-64.20) | | | 23.99 (22.79-28.02) | | ≤0.001 | | ≤0.001 |
| **CCL24** | 176.85 (115.66- 518.42) | 661.26 (357.91- 1003.77) | | | 207.12 (124.88-281.74) | | ≤0.001 | | ≤0.01 |
| **CCL25** | 380.41 (283.72-432.99) | 558.81 (421.87- 662.76) | | | 316.77 (270.06-345.53) | | ≤0.001 | | ≤0.001 |
| **CX3CL1** | 231.98 (164.365- 411.28) | 407.02 (366.85-576.72) | | | 158.38 (118.56-174.68) | | ≤0.001 | | ≤0.001 |
| **CXCL2** | 268.42 (187.28- 397.49) | 417.89 (272.62-559.7) | | | 199.75 (281.83-172.33) | | ≤0.001 | | ≤0.01 |
| **CXCL5** | 311.27 (98.06-741.31) | 1426.93 (729.78-2140.41) | | | 575.00 (332.67-990.96) | | ≤0.001 | | ≤0.001 |
| **CXCL6** | 42.39 (26.37- 57.02) | 61.23 (51.71-80.76) | | | 39.23 (28.9-54.415) | | ≤0.01 | | ≤0.01 |
| **CXCL11** | 145.75(31.21-402.13) | 46.74 (25.99-67.04) | | | 189.05 (160.16-213.18) | | ≤0.001 | | ≤0.01 |
| **IFNG** | 1.43 (1.05-35.63) | 49.75 (42.35-62.2) | | | 1.31 (1.22-1.42) | | ≤0.001 | | <0.001 |
| **IL-2** | 4.4 (2.95- 10.09) | 14.83 (12.3-17.97) | | | 4.17 (3.84-4.73) | | ≤0.001 | | ≤0.001 |
| **IL-4** | 7.23 (5.34- 24.81) | 26.53 (22.35-29.72) | | | 6.47 (5.82-7.33) | | ≤0.001 | | ≤0.01 |
| **MIF** | 2063.61 (724.05-4329.13) | 12089.63 (3528.79- 21472.19) | | | 1344.22 (564.61- 1825.88) | | ≤0.001 | | ≤0.01 |
| **TNF-A** | 13.2 (11.49- 19.16) | 21.44 (16.28- 27.36) | | | 11.15 (10.20-12.65) | | ≤0.001 | | ≤0.001 |
|  | | | | | | | | | |
| **Cytokine** | **TS-CONTROL**  **(pg/ml)** | | **BMS-CONTROL**  **(pg/ml)** | **TS-BMS-CONTROL**  **(pg/ml)** | | **Kruskal-Wallis**  **p value** | | **Mann-Whitney**  **[TS-CONTROL**  **Versus**  **BMS- CONTROL]**  **p value** | |
| **CX3CL1** | 355.36 (243.79- 397.98) | | 534.84 (392.46- 634.85) | 158.38 (118.56-174.68) | | ≤0.001 | | ≤0.001 | |
| **IL-1B** | 6.50 (3.68- 11.77) | | 2.85 (2.32- 3.62) | 6.8 (6.18-8.18) | | ≤0.01 | | ≤0.01 | |
| **IL-2** | 12.3 (5.82- 15.30) | | 15.78 (13.41- 20.24) | 4.17 (3.84-4.73) | | ≤0.001 | | ≤0.01 | |
| **IL-6** | 12.59 (10.22-13.81) | | 16.01 (12.59- 22.1) | 9.63 (8.52-10.17) | | ≤0.001 | | ≤0.01 | |

|  | **TS-COPD** | **TS-CONTROL** | **TS-BMS-CONTROL** | **Kruskal-Wallis** | **Mann-Whitney U** | **Spearman’s correlation** | | | | |
| --- | --- | --- | --- | --- | --- | --- | --- | --- | --- | --- |
|  | **(pg/ml)** | **(pg/ml)** | **(pg/ml)** | **p value** | **p value** | **FEV_1_** | | **FEV_1_/FVC** | | **COPD severity** |
| **CCL1** | 27.38 (24.26-44.77) | 50.87 (38.54-54.26) | 23.44 (25.95- 22.26) | ≤0.001 | ≤0.001 | ns | | r=0.365  p≤0.01 | | ns |
| **CCL7** | 22.18 (13.32-38.00) | 49.66(27.315-55.81) | 23.99(28.02-22.79) | ≤0.01 | ≤0.01 | ns | | ns | | ns |
| **CCL15** | 2979.58  ( 1016.3 -4740.20) | 8287.66 (5566.82-10180.96) | 3270.56 (4118.67-2526.68) | ≤0.01 | ≤0.001 | r=0.43  p≤0.01 | | r=0.419  p≤0.01 | | ns |
| **CCL17** | 69.05 (39.80-155.46) | 168.52 (111.65-205.07) | 155.01 (224.51-147.55) | ≤0.001 | ≤0.01 | ns | | ns | | ns |
| **CCL19** | 124.46 (58.29-196.73) | 204.90 (155.81-272.63) | 252.12 (281.37-211.52) | ≤0.01 | ≤0.01 | r=0.365  p≤0.01 | | r=0.363  p≤0.01 | | ns |
| **CXCL2** | 268.42(187.28-397.49) | 753.46 (290.37-857.49) | 199.75(281.82-172.32) | ≤0.01 | ≤0.01 | ns | | ns | | ns |
| **CXCL9** | 356.22 (239.24-518.25) | 601.80 (524.54-701.57) | 256.71(277.51-227.55) | ≤0.01 | ≤0.01 | ns | | ns | | ns |
| **IFN-G** | 1.43 (1.05-35.63) | 47.92 (28.52-59.58) | 1.31 (1.42-1.21) | ≤0.001 | ≤0.01 | ns | | ns | | r=-0.578  p≤0.01 |
| **MIF** | 2063.61 (724.04-4329.13) | 14115.77(2950.2-17033.25) | 1344.22(1825.87-564.61 | ≤0.001 | ≤0.01 | ns | | ns | | r=-0.613  p≤0.01 |
|  | **BMS-COPD** | **BMS-CONTROL** | **TS-BMS-CONTROL** | **Kruskal-Wallis** | **Mann-Whitney U** | **Spearman’s correlation** | | | | |
| **CCL15** | 5977.22 (1459.25-8646.12) | 8799.35 (5885.19-11715.51) | 3270.56 (4118.67-2526.68) | ≤0.001 | ≤0.01 | ns | ns | | ns | |
| **CCL27** | 1024.99 (743.09-1285.01) | 1409.14 (1179.38-1569.95) | 593.96 (516.40-1028.81) | ≤0.001 | ≤0.001 | ns | ns | | ns | |
| **CXCL13** | 26.03 (17.92-34.85) | 37.14 (24.24-44.64) | 22.77 (19.62-29.48) | ≤0.001 | ≤0.01 | ns | ns | | ns | |

**Supplementary table ST3:** Summarized representation of the significantly different serum chemokine and cytokine concentrations between TS-COPD versus TS-CONTROL and BMS-COPD versus BMS-CONTROL groups and their correlation to lung function and COPD severity. **Abbreviations:** **COPD:** Chronic obstructive pulmonary disease; **TS-CONTROL:** Tobacco smokers without COPD; **BMS CONTROL:** Biomass smoke exposed without COPD; **TS-BMS-CONTROL:** No Tobacco- and no biomass smoke exposed subjects; **FEV_1-post_:** Forced expiratory volume 1 second (post- bronchodilator challenge); **FVC:** Forced vital capacity. p<0.05 was considered as statistically significant, **ns:** not significant

Concentrations are represented as [median (25^th^-75^th^ percentile] pg/ml. p≤0.01 was considered as statistically significant.
